# Local variation in stress response of juvenile anadromous brown trout, *Salmo trutta*

**DOI:** 10.1002/ece3.11526

**Published:** 2024-06-25

**Authors:** Madeleine Berry, Lucas A. Zena, Jonathan A. C. Roques, Erik Sandblom, Eva B. Thorstad, Johan Höjesjö

**Affiliations:** ^1^ Department of Biological and Environmental Sciences University of Gothenburg Gothenburg Sweden; ^2^ SWEMARC, The Swedish Mariculture Research Center University of Gothenburg Gothenburg Sweden; ^3^ Norwegian Institute for Nature Research Trondheim Norway

**Keywords:** aerobic capacity, cortisol, hemoglobin, physiology, salmonid, spleen

## Abstract

Habitat fragmentation may cut off anadromous salmonids from parts of their potential native habitat and separate previously connected populations. Understanding the consequences of this is vital for fish management and prioritization of restoration activities. Here, we show that there is a significant difference in the body morphology, physiological stress response, and aspects contributing to aerobic capacity between juvenile anadromous brown trout, *Salmo trutta*, collected at a downstream site and an upstream site, separated by 2 km and several challenging stream sections, in a small unfragmented stream system in western Sweden. Following a standardized stress test, there were significant differences between fish from the upstream and downstream sites (plasma cortisol concentration, plasma osmolality, hematocrit, hemoglobin concentration, and mean corpuscular hemoglobin concentration). Plasma glucose concentration did not significantly differ between fish from the two sites. Fish from the upstream site had larger spleen mass, although there was no evidence of differences in ventricle mass or proportion of compact ventricular myocardium. These physiological differences indicate local variation in stress response and highlight the importance of considering local trait variation in river management. If a section of the river becomes fragmented or degraded, and there are differences in the juveniles in different parts of the river, the consequence for the population might be larger than the proportional loss of habitat.

## Graphical abstract

Stress response in juvenile anadromous brown trout was investigated to determine differences between individuals born upstream and downstream. Several organ morphology parameters relating to aerobic capacity were also assessed. Juveniles born upstream had larger spleens and a lower acute stress response compared with those born downstream.

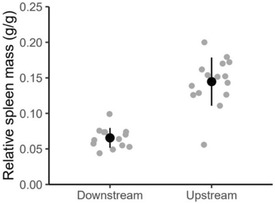

## 1 INTRODUCTION

Migratory fishes are threatened by numerous anthropogenic factors, such as climate change, habitat degradation, and fragmentation (Junge et al., 2014; Lin et al., 2017), all of which have the potential to restrict access to habitat and interfere with natural migration. Human infrastructure in rivers, particularly related to hydropower, creates migration barriers and reduces river connectivity. This results in large parts of the natural range becoming inaccessible and blocking of traditional migration routes for many species (Belletti et al., 2020). The separation of river sections caused by habitat fragmentation can result in differing environmental conditions and selective pressures experienced by individuals in the various sections. One example of a species that is affected by habitat degradation and fragmentation is brown trout, *Salmo trutta*. This species is widely distributed across Europe and important in both commercial and recreational fisheries as well as serving ecological and socio‐cultural functions (Blicharska & Rönnbäck, 2018; Liu et al., 2019; Mawle, 2018).

Anadromous brown trout migrate from freshwater systems to coastal areas as juveniles, and then to a large extent migrate back to their natal streams after a few months to 3 years as adults to spawn (Nevoux et al., 2019; Thorstad et al., 2016). Throughout their lifetime, they occupy many kilometers of river and individuals can be found throughout a river system (Cucherousset et al., 2005; Ferguson et al., 2019). Previous studies attribute the spatial distribution of juvenile fish within river stretches directly to maternal spawning site selection (Foldvik et al., 2010). The distance traveled upstream to spawn has been linked to many characteristics including the energetic cost of migration (Kristensen et al., 2011) and habitat type (Zimmer & Power, 2006). Adults compete for high‐quality spawning sites but will spawn in sub‐optimal redds when outcompeted or if adults are unable to complete longer distance migrations to higher quality sites (Zimmer & Power, 2006). Thus, spawning may occur throughout a river system with varying degrees of habitat quality. For instance, Saraniemi et al. (2008) found spawning sites ranging over 20 km in the river Oulankajoki, Northern Finland, and in large river systems, some sea trout may migrate several tens or hundreds of kilometers upstream, while others stay much closer to the estuary (Gabrielsen et al., 2021; Östergren et al., 2011). Even so, little is so far known about the phenotypic differences in sea trout migrating far and those undertaking shorter migrations.

A key aspect contributing to variation in adult upstream migration differences in other salmonids is aerobic capacity (Eliason et al., 2013; Eliason & Farrell, 2016; Lee et al., 2003). Aerobic capacity in fish refers to the ability of a fish species to utilize oxygen efficiently during aerobic metabolism. Fish species may exhibit specific physiological adaptations that enhance their aerobic capacity (Rosenfeld et al., 2020). Having greater aerobic capacity may allow adults to complete longer and more challenging migrations and potentially reaching better spawning sites. For example, different strains of adult sockeye salmon, *Oncorhynchus nerka*, with different migration route length and elevation exhibit considerable differences in cardiorespiratory physiology and aerobic capacity (Eliason et al., 2013). Traits such as heart size, compact myocardium thickness, and spleen size contribute to aerobic capacity. For instance, acclimation to seawater in rainbow trout, *Oncorhynchus mykiss*, results in higher proportions of compact myocardium compared with that of conspecifics in freshwater. This suggests an increased dependency on the coronary circulation, which provides oxygen‐rich blood to the compact myocardium, likely meeting the higher metabolic needs of osmoregulatory functions (Brijs et al., 2017; Wallbom et al., 2023). The salmonid heart is a highly phenotypically plastic organ and remodels across multiple levels of organization in response to environmental stimuli (Brijs et al., 2017; Gamperl & Farrell, 2004; Wallbom et al., 2023).

Response to environmental stressors is another important element to consider. For euryhaline migratory fishes, such as anadromous brown trout, the act of migration is associated with many stressors including physical exertion, osmoregulatory challenges, and increased predation risk (McCormick et al., 2013). Stress can be categorized into two main types: acute stress and chronic stress, depending on the duration and intensity of the stressor. The physiological response to stress is a complex endocrine process with many components and complex feedback loops, that have both short‐term effects such as increased ventilation rate (Brown et al., 2005) and long‐term effects such as lowered growth rate (Jentoft et al., 2005). Stress triggers a cascade of responses classified as primary, secondary, and tertiary responses (Barton, 2002). Primary responses are initiated by exposure to a stressor and include elevation of circulatory hormones, such as corticosteroids and catecholamines as well as activation of sympathetic pathways of the autonomic nervous system. Secondary responses then follow, of which some are triggered by these endocrine changes, such as hematological changes. Tertiary responses are typically more long term for instance growth inhibition (Wendelaar Bonga, 1997).

The most commonly used primary stress indicator in fish is plasma cortisol concentration (Barton, 2000; Seibel et al., 2021; Wendelaar Bonga, 1997). When exposed to a stressor, a common secondary stress response is splenic contraction and increases in the proportion of red blood cells in the blood via the release of stored erythrocytes. This elevation of red blood cells allows a fish to maximize its circulatory oxygen‐transporting capacity in times of need. Following stress, rainbow trout may release as much as 95% of their spleen erythrocyte reservoir, which alone may elevate hematocrit 23% and hemoglobin concentration 31% (Pearson & Stevens, 1991). Thus, hemoglobin concentration and hematocrit are also useful as indicators of stress (Jawad et al., 2004; Seibel et al., 2021). As well as an increase in the number of red blood cells, another common secondary response to stress is red blood cell swelling (Pearson & Stevens, 1991), increasing hematocrit and hemoglobin concentration while decreasing mean corpuscular hemoglobin concentration. Other secondary stress responses of interest are plasma glucose concentration and plasma osmolality (Seibel et al., 2021).

If aerobic capacity and stress response differ between individuals spawning at different sites along a stream, this should be reflected in differences in juveniles produced at different localities. Fish born at poorer quality sites are likely challenged by a sub‐optimal habitat and/or potentially parented by lower quality adults, which could lead to a scenario of spatial sorting of juvenile quality by genetics, maternal effects, or environmental conditions. Juvenile anadromous brown trout have limited dispersal prior to migration and one stream can therefore comprise multiple distinct groups distributed according to spawning sites (Beard & Carline, 1991; Quinn et al., 2012; Vera et al., 2010). Habitat alterations can lead to isolation or even local extinctions of sub‐populations and result in the loss of certain traits from the larger population (Junge et al., 2014; McClure et al., 2008). Thus, a better understanding of the spatial distribution of anadromous brown trout phenotypes could be used to highlight the importance of maintaining unfragmented stream ecosystems and aid conservation efforts directed at this economically and ecologically valuable resource (Butler et al., 2009; Gresh et al., 2000).

To our knowledge, there is no information on how juvenile anadromous brown trout differ in aerobic capacity or stress physiology along a river gradient. The overall aim of this study was, therefore, to investigate whether aspects contributing to aerobic capacity and stress responses differ in juvenile anadromous brown trout from upstream and downstream in a relatively small stream (<5 km long) where habitat quality is known to increase upstream. We predicted that fish originating upstream would have greater potential aerobic capacity, with larger hearts, greater proportion of compact myocardium, and larger spleens than fish originating downstream. Additionally, it was predicted that downstream fish would have a stronger response when exposed to a standardized stress challenge. This will be evidenced by an increase in plasma cortisol and glucose, elevated hematocrit, hemoglobin, plasma osmolality, and decreased mean corpuscular hemoglobin concentration for downstream fish compared with upstream fish.

## 2 METHODS

### 2.1 Study site

**FIGURE 1:**
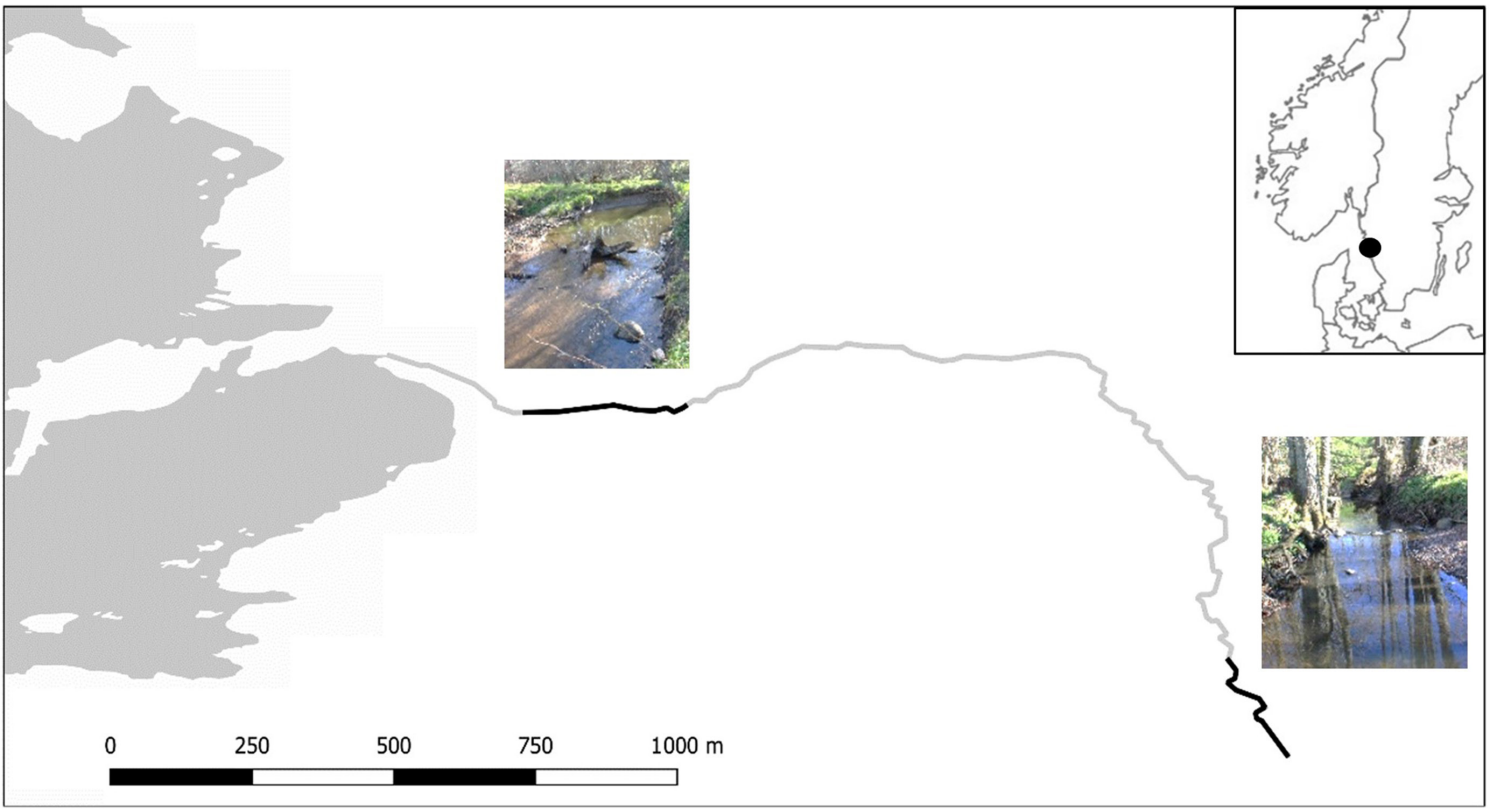
Map of the study site Haga å, in Southwestern Sweden. Black lines indicate electrofishing stretches.

The study was conducted in the stream Haga å in Västra Götaland, Sweden. Two study sites were selected, one 2700 m from the river mouth and one 300 m from the river mouth, both being approximately 100 m long (Figure 1). The stream is narrow (average width = 3 m) and relatively shallow (average depth = 0.3 m). The stretch between these two sites increases in elevation approximately 7 m and has multiple obstacles to migration both natural, such as waterfalls, and man‐made, such as culverts. At this stream, it is known that the population mostly consists of anadromous trout (Persson et al., 2023) and residents have rarely been found.

Fish were caught via electrofishing (Smith‐Root LR20B, Vancouver, Washington, USA) on September 29, 2021 at the upstream site (total caught = 82) and November 10, 2021 at the downstream site (total caught = 49). Of these, 15 individuals with 10–15 cm fork length were randomly selected at each site for use in this study (upstream: *n* = 15, mean length = 10.9 ± 0.5 cm; downstream: *n* = 15, mean length = 13.4 ± 1.1 cm), the remaining fish were used for an ongoing PIT tagging study. Approximately 300 m^2^ was electrofished at each site and each site was electrofished twice consecutively. During electrofishing, caught fish were held in the stream in boxes (60 cm × 40 cm × 40 cm) with small holes to allow waterflow through. Both sampling occasions were conducted at the same time of day (10:00–12:00) and under similar weather conditions.

### 2.2 Stress test and tissue sampling

Each fish was transferred into a 30 cm diameter bucket and chased for 5 min to exhaustion to exert a maximum acute stress response (Pearson & Stevens, 1991). The fish was then euthanized immediately using an overdose of benzocaine (ethyl 4‐aminobenzoate) (Sigma‐Aldrich, St. Louis, Missouri, USA), the effect of benzocaine was assumed to be similar in all individuals. Weight (g) and fork length (cm) were measured, and Fulton's condition factor (K) calculated for each fish using the equation weight (g)/length^3^ (cm) × 100. Blood samples were drawn immediately after death from the caudal vessels using a heparinized syringe and stored on ice. The spleen and ventricle were dissected out and stored on ice. Both the spleen and ventricle were blotted dry with tissue paper (Kimtech, Kimberly‐Clark Europe Limited, Surrey, UK), weighed, and relative masses were calculated using the equation raw organ mass/body mass × 100.

### 2.3 Myocardium tissue preparation and analysis

The ventricle samples were fixed in 4% paraformaldehyde (4% in phosphate‐buffered saline) at 4°C for 24 h and later transferred to 70% ethanol, prior to preparation for histological examination by standard paraffin wax techniques and staining with hematoxylin and eosin (see Zena et al., 2021 for details). Samples were scanned at 10× magnification (manual WSI scanning software, Microvisioneer, Esslingen am Neckar, Germany) and measurements of compact myocardium thickness and ventricle area were taken using imaging software Q path and ImageJ. The thickness of the compact myocardium layer was measured approximately every 100 μm around the perimeter of the ventricle with >40 measurements per sample. The mean of these measurements was divided by cross‐section area of the sample (Anttila et al., 2014). Three separate sections were measured for each individual ventricle in this way, and the mean taken from these sections was used to calculate compact myocardium thickness relative to the total ventricular area. Only samples with sufficient sample and image quality were analyzed (upstream *n* = 10, downstream *n* = 9).

### 2.4 Blood analyses

Blood samples were analyzed for hematocrit (Hct, %) and hemoglobin concentration ([Hb], g/dL). The Hct was determined as the fractional red cell volume after centrifugation of a subsample of blood in 80 μL heparinized microcapillary tubes at 10,000 rcf for 5 min in a Hct centrifuge (Haematokrit 210, Hettich, Tuttlingen, Germany). A handheld Hb 201+ meter (Hemocue® AB, Ängelholm, Sweden) was used to determine [Hb] in another blood subsample, and values were corrected for fish blood (Clark et al., 2008). Mean corpuscular hemoglobin concentration (MCHC, g/dL) was subsequently calculated as [Hb]/Hct × 100.

Following the hematological analyses, the remaining blood samples were centrifuged at 10,000 rcf for 5 min in a microcentrifuge (Eppendorf 5415D, Eppendorf, Hamburg, Germany). The plasma was collected and kept on ice during transportation to laboratory where it was kept frozen at −80°C until further analyses to determine the plasma Osmolality (mOsmol/L), alongside plasma glucose (mmol/L) and cortisol concentrations (ng/mL), one plasma sample was lost during transport. Plasma osmolality was measured using a cryoscopic osmometer with 50 μL as sample volume (Advanced Model 3320 Micro‐Osmometer4; Advanced Instruments Inc., Norwood, Massachusetts, USA), three plasma samples were not of sufficient size to analyze. The concentration of plasma glucose was determined in duplicates using a glucose assay kit (GAHK20, Sigma‐Aldrich, St. Louis, Missouri, USA). Plasma cortisol concentration was determined by a radioimmunoassay (RIA) in duplicates as previously described by Young (1986). Tritiated hydrocortisone‐[1,2,6,7‐3H(N)] (NET 396; NEN Life Sciences Products, Boston, Massachusetts, USA) was used as a tracer alongside cortisol antibody (Code: S020; Lot: 1014‐180,182, Guildhay Ltd., Guildford, Surrey, UK) validated by Sundh et al. (2011). Cortisol standards were prepared from hydrocortisone (Sigma, St. Louis, Missouri, USA), and radioactivity was determined with a Wallac 1409 liquid scintillation counter (LKB Instruments, Turku, Finland). Intra‐ and interassay coefficients of variation for this cortisol assay have been shown to be 3.9% and 5.4%, respectively, with a detection limit of 0.7 ng m/L (Sundh et al., 2011).

### 2.5 Statistical analyses

Statistical analyses were conducted using R Studio 1.0.153 (http://www.R‐project.org/). *T*‐tests were used to determine whether fish differed in length and weight between upstream and downstream sites. A correlation matrix was created using R packages corr and ggcorplot to visualize relatedness of response parameters (excluding proportion compact myocardium due to the small sample size) (Appendix). One fish was excluded for relative spleen and ventricle masses due to suspected measurement errors. Normality was checked using Shapiro–Wilks tests, which showed relative spleen and ventricle masses were non‐normally distributed. A MANOVA was conducted with site as the independent variable, weight as a covariate, and blood parameters (hematocrit, hemoglobin concentration, MCHC, plasma cortisol concentration, plasma glucose concentration, and plasma osmolality) as well as K as response variables, four individuals were excluded due to missing data values. This showed the site to be a significant factor (Site: *F*_1,17_ = 16.8, *p* < .001; weight: *F*_1,17_ = 1.0, *p* = .5), and subsequently, ANCOVA tests with type III errors were conducted on each parameter (hematocrit, hemoglobin concentration, MCHC, plasma cortisol concentration, plasma glucose concentration, plasma osmolality, condition, and proportion compact myocardium) using R package car. Relative spleen and ventricle masses were analyzed using non‐parametric Kruskal–Wallis tests.

## 3 RESULTS

### 3.1 Body and organ morphology

**FIGURE 2:**
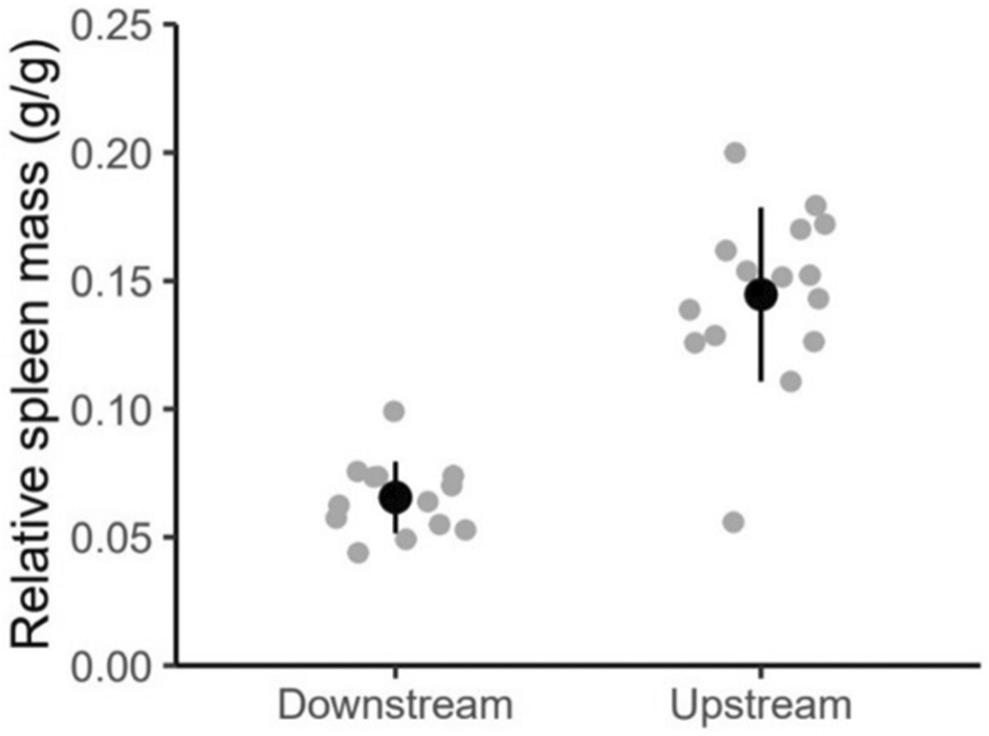
Spleen mass (accounting for body mass) of downstream and upstream fish, spleen mass is significantly lower in downstream fish (*p* < .05). Means are displayed with solid black circles. Error bars show mean ± standard deviation. Individual data points are displayed in gray circles.

Fish from the downstream site were longer and heavier (mean length = 13.4 ± 1.1 cm, mean weight = 25.4 ± 7.0 g) than upstream fish (mean length = 10.9 ± 0.5 cm, mean weight = 14.1 ± 2.1 g) (*t* = 7, df = 18, *p* < .001; *t* = 6, df = 16, *p* < .001). K did not differ between the two sites (*F*_1,27_ = 3.3, *p* = .08) and did not differ with body mass (*F*_1,27_ = 1.1, *p* = .3). Kruskal–Wallis tests showed that relative spleen mass was lower in downstream fish compared with upstream fish (Figure 2) (df = 1, *χ* ^2^ = 17.2, *p* < .001), and there was no difference found in relative ventricle mass between the two sites (df = 1, *χ* ^2^ = 2.5, *p* = .1). Proportion compact myocardium did not differ between fish from the two sites (*F*_1,16_ = 3.9, *p* = .07) and was related to body mass (*F*_1,16_ = 6.8, *p* = .02).

### 3.2 Blood parameters

**FIGURE 3:**
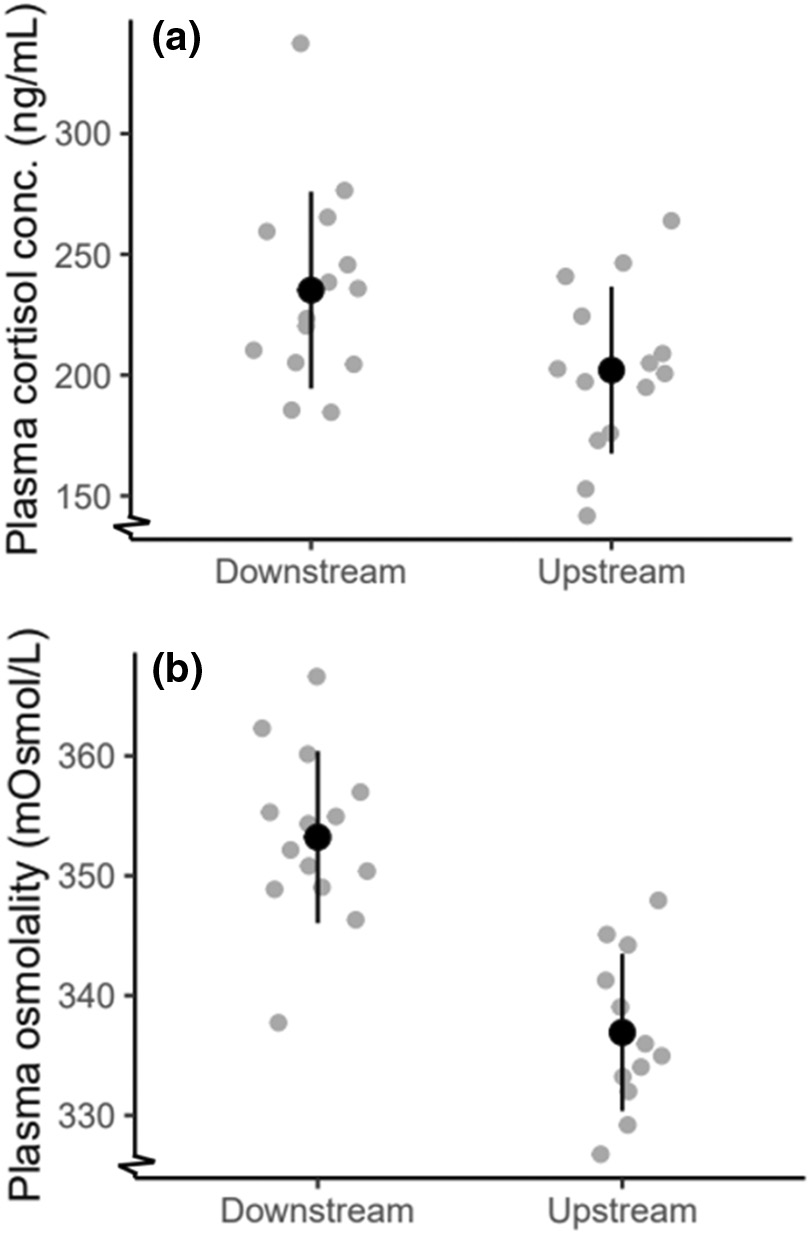
Panel (a) shows the plasma cortisol concentration of downstream and upstream fish, downstream fish had significantly higher plasma cortisol (*p* < .05). Panel (b) shows the plasma osmolality of downstream and upstream fish, downstream fish had significantly higher plasma osmolality (*p* < .05). Means are displayed with solid black circles. Error bars show mean ± standard deviation. Individual data points are displayed in gray circles.

Plasma cortisol concentration (ng/mL) was higher in downstream than upstream fish (Figure 3a) (*F*_1,26_ = 5.7, *p* = .02) and did not differ with body mass (*F*_1,26_ = 0.7, *p* = .4) Plasma glucose concentration (mmol/L) did not differ between the two sites (upstream mean = 9.8 ± 2.2, downstream mean = 11.3 ± 1.8) (*F*_1,26_ = 4, *p* = .9), or body mass (*F*_1,26_ = 4, *p* = .07). Plasma osmolality (mOsmol/L) was higher in downstream than upstream fish (Figure 3b) (*F*_1,23_ = 36, *p* = .003) and did not differ with body mass (*F*_1,23_ = 0.5, *p* = .5).

**FIGURE 4:**
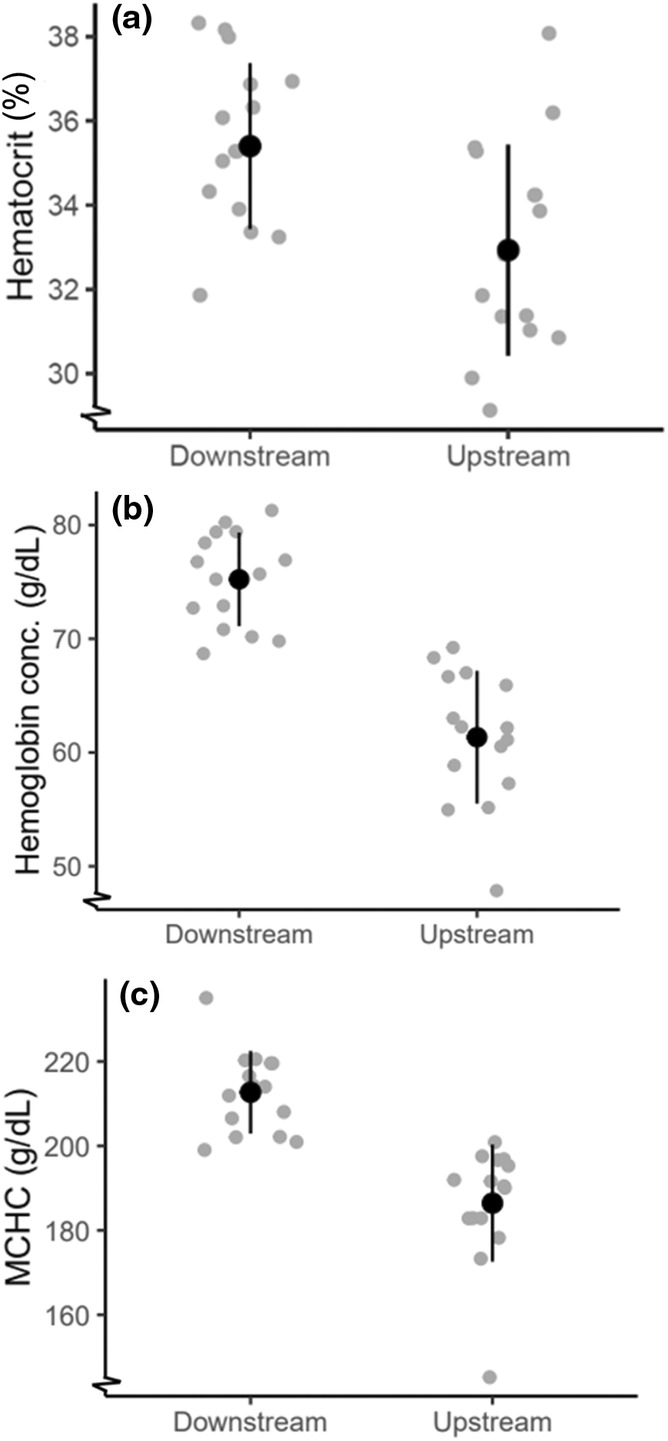
Panel (a) shows hematocrit levels of downstream and upstream fish, downstream fish had a significantly greater proportion of hematocrit (*p* < .05). Panel (b) shows hemoglobin concentration of downstream and upstream fish, downstream fish had significantly higher hemoglobin concentration (*p* < .05). Panel (c) shows mean corpuscular hemoglobin concentration, downstream fish had significantly higher MCHC (*p* < .05). Means are displayed with solid black circles. Error bars show mean ± standard deviation. Individual data points are displayed in gray circles.

Hematocrit was higher in downstream fish (Figure 4a; *F*_1,27_ = 9, *p* = .03) and did not differ with body mass (*F*_1,27_ = 0.3, *p* = .6). Hemoglobin concentration was higher in downstream than upstream fish (Figure 4b) (*F*_1,27_ = 18.8, *p* < .001). MCHC was higher in downstream than upstream fish (Figure 4c) (*F*_1,27_ = 7.8, *p* = .01) and did not differ with body mass (*F*_1,27_ = 3.2, *p* = .09).

## 4 DISCUSSION

Our results suggest there are differences in organ and blood variables underlying aerobic capacity and stress response in juvenile anadromous brown trout between different locations, even within a relatively small stream system. Fish from the upstream site were smaller and had larger spleens. When exposed to a standardized stress test, fish from the downstream site had higher plasma cortisol concentration, plasma osmolality, hematocrit, hemoglobin concentration, and MCHC, suggesting a greater response to an acute stressor. Local variation in migratory fish within a stream can arise in several ways: spatial distribution, parental characteristics related to migration distance, and local variation in environmental conditions.

High plasma cortisol concentrations are indicative of a high acute primary stress response and plasma cortisol concentration was higher in fish from the downstream than upstream site. Elevated plasma glucose has been seen in Atlantic salmon, *Salmo salar*, subjected to both short‐ and long‐term handling stress (Fast et al., 2008), however, there was no significant difference between groups in the present study. While cortisol can promote the release of glucose into the bloodstream, it can also enhance glucose utilization in various tissues, and previous studies have suggested plasma glucose concentrations can be challenging to interpret in the context of stress (Flodmark et al., 2002; Mommsen et al., 1999).

Spleen contraction occurs following stress, releasing stored erythrocytes to increase hematocrit and hemoglobin concentration thereby maximizing oxygen‐carrying capacity (Pearson & Stevens, 1991; Wells et al., 1989). A larger spleen size could indicate a greater inherent capacity to elevate blood oxygen‐carrying capacity via splenic contraction. While aerobic capacity has not been directly measured in the present study, larger spleens in upstream fish could contribute to a potentially greater aerobic capacity. Greater aerobic capacity has been linked to upstream migration distance in adult salmonids (Eliason et al., 2013) and downstream migration distance in smolts via a positive correlation with hematocrit (Vainikka et al., 2023). It may therefore be beneficial for juveniles from upstream sites to have greater aerobic capacity, when undergoing a long migration toward the sea compared with juveniles from downstream sites.

Increased hematocrit can result from a combination of both elevated erythrocyte release and cell swelling. Adrenaline released during stress induces red blood cell swelling, which increases blood oxygen affinity (Nikinmaa, 1982). Here, we see higher hematocrit and hemoglobin concentration and less cell swelling (lower mean corpuscular hemoglobin concentration) in downstream fish compared with upstream fish. Greater hematocrit and hemoglobin concentration could also contribute to aerobic capacity; however, given baselines could not be established, it is difficult to disentangle inherent aerobic capacity and rises in red blood cells associated with response to stress. Plasma osmolality was 5% higher in downstream fish compared with upstream fish, which could be due to osmotic stress associated with the downstream site where salinity is thought to be slightly higher.

Contrary to our expectations, neither ventricle mass (accounting for body mass) nor proportion of compact myocardium differed between the two groups indicating similar cardiac output in fish from both sites. During analysis of the ventricle samples, it became apparent that most if not all individuals at both sites were suffering from mild pericarditis. The ultimate cause of this inflammation is unknown at this time but can be associated with a viral infection in diseases such as cardiomyopathy syndrome, heart, and skeletal inflammation and pancreas diseases (Kongtorp et al., 2004), all commonly found in farmed salmonids such as Atlantic salmon and rainbow trout, but also identified in wild salmonid populations (Garseth et al., 2012). The presence of the virus causing heart and skeletal inflammation was confirmed in wild resident brown trout in the Czech Republic (Pojezdal et al., 2020) and wild anadromous brown trout in Norway (Madhun et al., 2016), although none of the positive fish for the virus showed gross heart pathologies typically associated with the disease. Therefore, further investigation is needed to determine whether pericarditis found in the present study is associated with any of the viral diseases causing heart inflammation in salmonids and whether it can impact the cardiac performance and ability to cope with stress in wild anadromous brown trout.

Although it is appropriate to respond to stress in the right circumstances, it is also a trade‐off. The response should not be too costly to produce or too costly to reverse once the stressor is removed and should have the desired effect of surviving the stressor. From a physiological standpoint, primary and secondary stress responses are likely adaptive and help animals maintain homeostasis under challenging conditions. Problems mainly arise when stress becomes chronic, which may lead to detrimental tertiary stress responses (e.g., reduced growth and disturbed reproduction). There is a metabolic cost to a stress response and negative effects associated with certain aspects. For example, increasing hemoglobin concentration also increases blood viscosity, and subsequently, the energy needed to circulate blood, and in the long term, this can lead to myocardial interstitial fibrosis (Clark & Rodnick, 1999).

Now, we have established there are significant differences in a number of parameters between fish found upstream and downstream, but what is the driving force? There are several possible mechanisms of interest: habitat, displacement, or parentage/genetic contribution or a combination of these could be responsible. Although the stream is largely similar throughout it is known that habitat quality for trout increases upstream in this system. The upstream site is a good quality trout habitat; gravel substrate (average particle diameter 0.2–2 cm), average depth 30 cm, and an abundance of pool riffle structure. The downstream site is arguably a poorer quality trout habitat, having a finer substrate (average particle diameter 0.02–0.2 cm), deeper water (mean = 40 cm), and fewer pool/riffle structures. Generally, most rivers differ in a longitudinal gradient where slope is higher, gravel structure more coarse, and allochthonous input higher upstream in comparison to downstream sites. This usually makes the upstream habitat more suitable for salmonid fishes with better spawning grounds and better and more stable oxygen conditions (Vannote et al., 1980). In agreement, habitat quality of this stream increases along a gradient from downstream to upstream stretches. However, the spatial distribution of habitat quality can differ among rivers and streams, for example, due to fragmented systems with naturally existing lakes, inflow from groundwater, or anthropogenically introduced changes such as dams (Doretto et al., 2020; Jones, 2010), and therefore, it may not always be the case that upstream sites are of higher quality.

The role of habitat is also relevant when considering the displacement of individuals. If upstream habitat is more desirable, then dominant individuals outcompeting subordinates can result in their displacement downstream. In this case, there is the possibility fish originated upstream and subsequent “losers” have been forced to the downstream site. However, there are known successful spawning sites at both sites thus we know individuals are being produced in both areas and the distance between the sites are relatively long with multiple natural and man‐made obstacles making the displacement theory less likely. In support, juvenile anadromous brown trout have limited dispersal within a stream (Höjesjö et al., 2015; Palm et al., 2023; Vøllestad et al., 2012) and generally stay in the area in which they are born prior to migration (Quinn et al., 2012). Thus, where a juvenile is found could be considered approximate to the distance migrated upstream by its parents. Juveniles at the downstream site are likely parented by short‐distance migrants and fish at the upstream site by long‐distance migrants. In the future, it is important to examine any differences between sites at different year classes to determine whether the effect is stronger at an earlier stage.

Migration is an arduous process, and longer migrations are more challenging (Jonsson & Jonsson, 2006); possessing certain characteristics may influence how far individuals can successfully travel. Robust, physically fit individuals will be better suited to cover distance and traverse obstacles encountered during long freshwater migrations. These obstacles could impede other individuals and act as a barrier to further migration. Given the energy expended during migration, it is logical to expect certain physiological aspects to differ between individuals with different migratory strategies (Boel et al., 2014). These differences could then be reflected in the offspring, effects from parents could be both genetic and/or a maternal effect of energy transfer to eggs (Höjesjö et al., 2011). It is worth noting relatedness of individuals was not tested in this study. We may expect fish originating from the same site are more likely to have some degree of relatedness and therefore this could also contribute to the differences seen here and is an avenue for further study.

Stress is a critical influence on any animal's life, and it is important to understand how individuals differ in their response to acute stressors. Downstream fish had relatively higher plasma cortisol and varied in other blood parameters relevant to stress compared with their counterparts upstream. We have shown size and physiological condition of juvenile anadromous brown trout may vary between different sections of a watershed. Finding these differences even at a small spatial scale highlights the need to take local trait variation into account when considering management, such as prioritization of restoration sites. A strong suggestion for future research is to compare the productivity and quality of juveniles originating from different sections within streams given the likely variation in migration mortality with varying outward migration distances. This then needs to be examined on a full life cycle; how does the origin of offspring correlate to fitness such as survival in the sea, growth, and size at maturation? It may be beneficial to maintain a variation of phenotypes to better cope with future anthropogenic pressure such as climate change, for example, drought conditions during heat waves for are more likely to be detrimental for juvenile salmonids at upstream sites. In summary, we have shown preserving access to upstream sites or higher quality sections is key to maintaining local variation and the production of individuals with a lower acute stress response.

## AUTHOR CONTRIBUTIONS

**Madeleine Berry:** Conceptualization (supporting); data curation (lead); formal analysis (lead); investigation (equal); methodology (equal); project administration (lead); writing – original draft (lead); writing – review and editing (equal). **Lucas A. Zena:** Investigation (equal); methodology (equal); writing – review and editing (equal). **Jonathan A. C. Roques:** Investigation (equal); methodology (equal); writing – review and editing (equal). **Erik Sandblom:** Conceptualization (equal); methodology (equal); resources (equal); supervision (equal); writing – review and editing (equal). **Eva B. Thorstad:** Conceptualization (equal); supervision (equal); writing – original draft (equal); writing – review and editing (equal). **Johan Höjesjö:** Conceptualization (equal); funding acquisition (lead); methodology (equal); project administration (equal); resources (lead); supervision (lead); writing – original draft (equal); writing – review and editing (equal).

## CONFLICT OF INTEREST STATEMENT

The authors declare no conflict of interest.

## Supporting information


Data S1.

## Data Availability

Data are provided as Data S1.

## 1

Correlation matrix of response variables, a degree of correlation is expected between response variables as they are all related to stress.
